# Molecular Characteristics of Colistin Resistance in *Acinetobacter baumannii* and the Activity of Antimicrobial Combination Therapy in a Tertiary Care Medical Center in Lebanon

**DOI:** 10.3390/microorganisms12020349

**Published:** 2024-02-08

**Authors:** Antoine Abou Fayad, Louis-Patrick Haraoui, Ahmad Sleiman, Hadi Hussein, Frédéric Grenier, Ghada Derbaj, Dana Itani, Sereen Iweir, Nour Sherri, Wael Bazzi, Sari Rasheed, Arax Tanelian, Mariam Miari, Bassam el Hafi, Souha S. Kanj, Zeina A. Kanafani, Ziad Daoud, George F. Araj, Ghassan M. Matar

**Affiliations:** 1 Department of Experimental Pathology, Immunology and Microbiology, Faculty of Medicine, American University of Beirut, Beirut 1107 2020, Lebanon; aa328@aub.edu.lb (A.A.F.); ahmad.sleiman@uclouvain.be (A.S.); ghadaderbaj6@outlook.com (G.D.); di13@aub.edu.lb (D.I.); siweir@crdfglobal.org (S.I.); nas51@mail.aub.edu (N.S.); wmb11@mail.aub.edu (W.B.); sari.rasheed@helmholtz-hips.de (S.R.); aat24@mail.aub.edu (A.T.); msm50@mail.aub.edu (M.M.);; 2 Center for Infectious Diseases Research, American University of Beirut, Beirut 1107 2020, Lebanon; sk11@aub.edu.lb (S.S.K.); zk10@aub.edu.lb (Z.A.K.); garaj@aub.edu.lb (G.F.A.); 3 World Health Organization (WHO) Collaborating Center for Reference and Research on Bacterial Pathogens, Beirut 1107 2020, Lebanon; 4 Department of Microbiology and Infectious Diseases, Faculty of Medicine and Health Sciences, Université de Sherbrooke, Sherbrooke, QC J1K 2R1, Canada; louis.patrick.haraoui@usherbrooke.ca; 5 Centre de recherche Charles-Le Moyne, Hôpital Charles-Le Moyne, Greenfield Park, QC J4V 2G9, Canada; 6 Department of Biology, Faculty of Science, Université de Sherbrooke, Sherbrooke, QC J1K 2R1, Canada; frederic.grenier@usherbrooke.ca; 7 Division of Infectious Diseases, Department of Internal Medicine, Faculty of Medicine, American University of Beirut, Beirut 1107 2020, Lebanon; 8 Laboratory Department, My Michigan Health Midland Medical Center, College of Medicine, Central Michigan University, Saginaw, MI 48602, USA; daoud1z@cmich.edu; 9 Department of Pathology and Laboratory Medicine, Faculty of Medicine, American University of Beirut, Beirut 1107 2020, Lebanon

**Keywords:** antimicrobial resistance, *Acinetobacter baumannii*, colistin resistance

## Abstract

(1) Background: Infections with pan-drug-resistant (PDR) bacteria, such as *A. baumannii*, are becoming increasingly common, especially in healthcare facilities. In this study, we selected 15 colistin-resistant clinical *A. baumannii* isolates from a hospital in Beirut, Lebanon, to test combination therapies and determine their sequence types (STs) and the mechanism of colistin resistance using whole-genome sequencing (WGS). (2) Methods: Antimicrobial susceptibility testing via broth microdilution against 12 antimicrobials from different classes and growth rate assays were performed. A checkerboard assay was conducted on PDR isolates using six different antimicrobials, each in combination with colistin. Genomic DNA was extracted from all isolates and subjected to WGS. (3) Results: All isolates were resistant to all tested antimicrobials with the one exception that was susceptible to gentamicin. Combining colistin with either meropenem, ceftolozane–tazobactam, or teicoplanin showed synergistic activity. Sequencing data revealed that 67% of the isolates belonged to Pasteur ST2 and 33% to ST187. Furthermore, these isolates harbored a number of resistance genes, including *bla*_OXA-23_. Mutations in the *pmrC* gene were behind colistin resistance. (4) Conclusions: With the rise in antimicrobial resistance and the absence of novel antimicrobial production, alternative treatments must be found. The combination therapy results from this study suggest treatment options for PDR ST2 *A. baumannii*-infected patients.

## 1. Introduction

The rising emergence of multidrug-resistant Gram-negative pathogens, especially carbapenem-resistant ones, poses a significant public threat worldwide [1]. Carbapenem resistance in *Acinetobacter baumannii* has been reported in Lebanon and several other countries in the Eastern Mediterranean region [2]. Therapeutic options against the emerging pathogens are hampered by the rise in resistance to almost all available classes of antimicrobials except tigecycline and colistin/polymixins, which are considered last-resort antimicrobials [3]. Colistin confers its main mode of action by binding to the negatively charged regions of the hydrophilic lipid A component of the lipopolysaccharide (LPS) component of the Gram-negative bacterial cell wall, causing the destabilization of the cell membrane and consequently cell lysis and death [4].

The increase in the usage of colistin accompanied by its uncontrolled use in agriculture globally have contributed to the dissemination of colistin-resistant Gram-negative pathogens worldwide [5]. Colistin resistance is mainly attributed to the decrease in the affinity of colistin to its target, either by the phosphoethanolamine modification of LPS by the two-component system, *pmrAB*, [6] or by the loss of LPS due to mutations in lipid biosynthesis genes [7]. The discovery of *Escherichia coli* in humans and animals harboring the plasmid-mediated colistin-resistance *mcr-1* gene provides a further mechanism for the rapid dissemination of colistin resistance and significantly limits the therapeutic options for infected patients [8].

Combination therapy can offer a better treatment option than monotherapy for the treatment of extensively drug-resistant Gram-negative pathogens [9]. For instance, the synergistic effect of colistin in combination with carbapenems and aminoglycosides on *A. baumannii* is assessed [10]. The aim of this study is to investigate the in vitro activity of six different antimicrobial combination therapies against colistin-resistant *A. baumannii* isolates and to determine, at the molecular level, the Pasteur STs and the mechanism of colistin resistance in *A. baumannii*.

## 2. Materials and Methods

### 2.1. Source of Isolates

A total of 15 non-consecutive colistin-resistant *A. baumannii* isolates were collected in 2018 from the clinical microbiology laboratory in the Pathology and Laboratory Medicine department at the American University of Beirut Medical Center. Ethical approval was obtained from the American University of Beirut Institutional Review Board (AUB-IRB) committee and patient data were coded to eliminate any link between sample identifiers and patient identities.

### 2.2. Identification of Isolates

The recovered isolates in culture were identified using the Matrix-Assisted Laser Desorption/Ionization Time of Flight (MALDI-TOF) system (Bruker Daltonik, GmbH, Bremen, Germany) with a score of green flags [11].

### 2.3. Antimicrobial Susceptibility Testing

Antimicrobial susceptibly testing was performed by broth microdilution against 12 different antimicrobials from different antimicrobial classes. A serial dilution was performed with concentrations ranging from 1024 µg/mL to 1 µg/mL and the plate was incubated at 37 °C for 18–24 h. All the experiments were run in duplicates. The results were interpreted according to the CLSI M100 guideline [12,13]. Control strains *Escherichia coli* ATCC^®^ 25922 and *Pseudomonas aeruginosa* ATCC^®^ 27853 were used in parallel to monitor the MIC results. Isolates susceptible to one or more antimicrobial agent from one or two different classes were considered extensively drug-resistant (XDR), while those resistant to all classes, including polymyxins, were considered pan drug-resistant (PDR).

### 2.4. Antimicrobial Combination Therapy

Antimicrobial combination therapy was evaluated with colistin combined separately with tigecycline, levofloxacin, amikacin, meropenem, ceftolozane/tazobactam, and teicoplanin. The highest concentration of each antibiotic was chosen based on the direction of the serial dilution: 4 × MIC for each antibiotic diluted in the horizontal direction and 3 × MIC for each antibiotic diluted vertically. Colistin was always fixed in the horizontal direction where wells A1 to A10 were set to test for the MIC value while wells B1-H1 to B10-H10 were used for combination testing. For the other antibiotics that were serially diluted in the vertical direction, B11-H11 were set to test the MIC value of the antibiotic, while wells B1-B10 to H1-H10 were used for combination testing. Wells A12-D12 served as a positive control while wells E12-H12 served as a negative control. After the addition of 10 μL of the 5 × 10^6^ CFU/mL bacterial suspension, a total volume of 100 μL was achieved in all the wells, and the plate was then placed in the incubator at 37 °C for 18–24 h. The results were interpreted by the calculation of the Fractional Inhibitory Concentration Index (FICI). The ΣFIC = FIC‘A’ + FIC‘B’, where FIC A was the MIC of drug A in the combination/MIC of drug A alone, and FIC B was the MIC of drug B in the combination/MIC of drug B alone. The combination was considered synergistic when the ΣFIC index was ≤0.5, indifferent when the ΣFIC index was >0.5 to ≤4, and antagonistic when the ΣFIC was >4 [14].

### 2.5. Growth Rate Assay

To determine the growth rates of the isolates, freshly plated cultures were first subcultured on MacConkey agar and incubated at 37 °C for 18–24 h. The following day, a loopful of each bacterial isolate was transferred into 10 mL of sterile cation-adjusted Mueller–Hinton broth and incubated at 37 °C for 18–24 h. Then, the turbid inoculated broth of each isolate was diluted at 1:1000, and then 200 µL aliquots were transferred into 4 separate wells of a 96-well microtiter plate. The replication rate of each tested isolate was measured using a plate reader (OD 600 nm) for 16 h with readings at 30 min intervals. The results were then averaged, normalized, and plotted against the *A. baumannii* DSM^®^ 30008 [15].

### 2.6. Serial Passaging of Colistin-Resistant Isolates

For each isolate, a single colony was inoculated in 2 mL of LB broth and incubated at 37 °C for 18–24 h. Serial passaging with antibiotic-free LB broth was performed for 7 days, followed by the reassessment of the MICs of these isolates against colistin [16].

### 2.7. Whole-Genome Sequencing (WGS)

All isolates underwent short-read sequencing. DNA libraries were prepared from extracted gDNA using the NEBNext Ultra II FS DNA Library Prep Kit for Illumina (Illumina, San Diego, CA, USA). The DNA was purified and size selected using Ampure XP beads (Beckman Coulter, Brea, CA, USA) and quantified using the Quant-it PicoGreen dsDNA assay (Thermo Fisher, Waltham, MA, USA). The quality and size distribution of the DNA were assessed on a fragment analyzer using the HS NGS Fragment Kit (Agilent, Santa Clara, CA, USA). The pooled samples were then sequenced on a NovaSeq6000 (Illumina, San Diego, CA, USA) as PE250.

All strains also underwent long-read DNA sequencing. Extracted gDNA was treated with the NEBNext Ultra II End Repair/dA-Tailing Module (NEB). Then, barcodes from the Native Barcoding Expansion 1–12 and 13–24 from Oxford Nanopore Technologies (Oxford Nanopore Technologies, Oxford, UK) were ligated using the NEBNext Ultra II Ligation Module (NEB). The DNA was purified using Ampure XP beads (Beckman Coulter). The DNA from different barcoded samples was pooled and the adapter AMII (ONT) was ligated using the NEBNext Ultra II Ligation Module (NEB). Sequencing was performed with an R10.4 MinION Flow Cell using a MinION Mk1B (ONT). All the sequences were deposited in the NCBI under project number PRJNA979211.

### 2.8. Bioinformatics Analysis of the Isolates

For the Illumina reads, a quality assessment and trimming were performed using fastp 0.21.0 with—cut_right—cut_window_size 4—cut_mean_quality 20—length_required 30—detect_adapter_for_pe [17]. Samples with less than a 20X coverage were resequenced. Assemblies were created using Unicycler 0.4.9 [18] using the trimmed Illumina short reads and ONT long reads when available. Contigs were filtered to retain only those above 500 bp.

Taxonomic identifications were performed for the assemblies using Kraken 2 (2.0.9-beta) [19].

Antibiotic-resistance genes were found using ResFinder 3·0 [20]. Detected blaOXA variants were curated using BLDB [21]. Two distinct multi-locus sequence typing (MLST) schemes exist for *A. baumannii*, known as Oxford and Institut Pasteur [22], with the latter being used in this study. The STs of each strain were determined using mlst 2.11 (Seemann T, mlst Github https://github.com/tseemann/mlst, accessed on 8 April 2022), which made use of the PubMLST website (https://pubmlst.org/, accessed on 8 April 2022) [23]. Assemblies were annotated with Prokka 1.14.5 [24] using the additional databases Pfam, TIGRFAM, and the blaOXA variants present in the BLDB [21].

## 3. Results

### 3.1. Antimicrobial Susceptibility Testing

Broth microdilution results show that all the isolates are resistant to meropenem, imipenem, ceftazidime, cefepime, ciprofloxacin, levofloxacin, colistin, amikacin, tetracycline, piperacillin/tazobactam, and trimethoprim/sulfamethoxazole, and 14 (93%) to gentamicin. Out of the 15 isolates, 1 (7%) is extensively drug-resistant and 14 (93%) are pan-drug-resistant (Table 1).

### 3.2. Antimicrobial Combination Therapy

Antimicrobial combination therapy was performed using six different combinations. The results show synergistic activity for the colistin/meropenem, colistin/ceftolozane–tazobactam, and colistin/teicoplanin, and an indifferent activity for the combinations of colistin/tigecycline and colistin/amikacin in all the 15 *A. baumannii* isolates. However, for the colistin/levofloxacin combination, 13 (87%) and 2 (13%) isolates showed synergistic and indifferent activities, respectively.

### 3.3. Serial Passaging of Colistin-Resistant Isolates

After 7 days of serial passages in antibiotic-free LB broth, the broth microdilution results showed that no change in the MIC of colistin occurred in all the 15 colistin-resistant *A. baumannii* isolates.

### 3.4. Growth Rate

**Figure 1:**
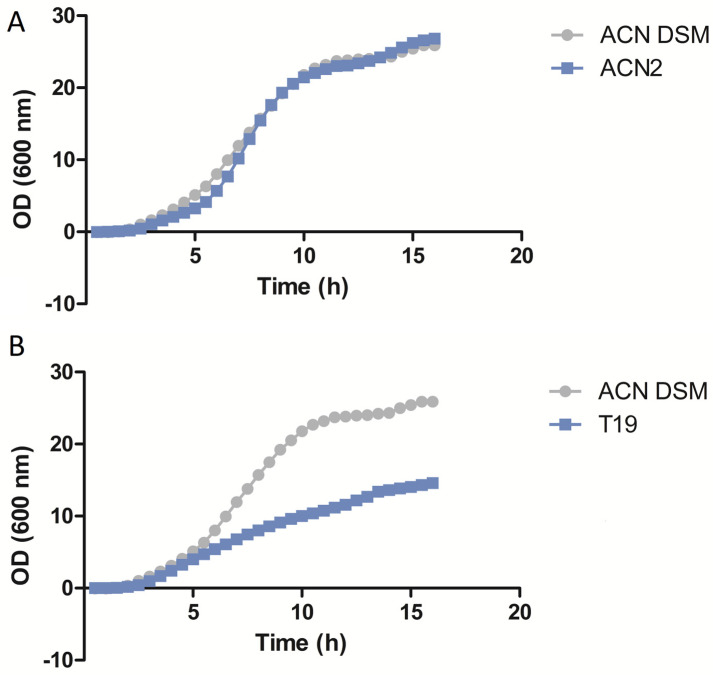
Growth rate assay results of isolates ACN2 (**A**) and T19 (**B**) compared to *A. baumannii* DSM 30008.

The growth rate results reveal that 13 (87%) isolates have similar growth rates when compared to the wild-type strain *A. baumannii* DSM^®^ 30008 (Figure 1A and Figures S1–S3). However, two (13%) isolates had lower growth rates when compared to the wild-type strain *A. baumannii* DSM^®^ 30008 (Figure 1B and Figure S4).

### 3.5. Whole-Genome Sequencing

Whole-genome sequencing results show that 10 (67%) and 5 (33%) isolates belong to sequence types (ST) 2 and 187, respectively. Moreover, a plethora of antimicrobial-resistance (AMR) genes that encoded resistance to β-lactams, aminoglycosides, sulfonamides, macrolides, and tetracyclines were detected among the 15 *A. baumannii* isolates. They were distributed as follows: bla_OXA-23_ (*n* = 8), bla_OXA-225_ (*n* = 1), bla_ADC-25_ (*n* = 9), bla_TEM-1D_ (*n* = 8), aph(3′)-VIa (*n* = 2), aph(3′)-Ia (*n* = 8), armA (*n* = 8), aph(6)-Id (*n* = 9), aph(3″)-Ib (*n* = 9), sul2 (*n* = 9), mph(E) (*n* = 14), msr(E) (*n* = 14), and tet(B) (*n* = 14) (Table 2).

Resfinder on CGE was the first resort to determine the molecular determinant of colistin resistance in the 15 *A. baumannii* isolates. However, no AMR genes were found that could explain this resistance. Then, a BLAST search for the six variants (pmrA, pmrB, pmrC, lpxA, lpxC, and lpxD) associated with colistin resistance in *A. baumannii* was performed. The BLAST search results show that a mutation in the pmrC variant is the reason for colistin resistance in 14 (93%) out of the 15 *A. baumannii* isolates. The mutations were G326A and R109H and C950T and A317V in 13 (87%) and 1 (6%) isolates, respectively. The reason for colistin resistance in isolate T19 remains unexplained (Table 3).

## 4. Discussion

Among six areas, each falling under a WHO regional office and together encompassing a total of 41 countries, the Eastern Mediterranean region had the second highest prevalence of colistin-resistant *A. baumannii* clinical isolates according to a 2020 meta-analysis that covered studies between the years 2000 and 2017 [25]. Moreover, Lebanon recorded the highest prevalence (17.5%) of all countries included in the study [25]. Similarly, a retrospective study that collected data from 16 hospitals between 2011 and 2013 in Lebanon found that *A. baumannii* showed a 17.1% resistance to colistin [26]. The occurrence of colistin resistance in *A. baumannii* was also monitored through studies conducted in other countries in regions such as Egypt, Iraq, Saudi Arabia, and Iran. These studies reported different ranges of prevalence of colistin-resistant *A. baumannii*, and hence indicated a diverse and alarming occurrence of colistin-resistant *A. baumannii* in the region, which highlights the urgent need for treatment options that circumvented resistance to last-resort antimicrobials, such as combination therapies. To that end, studies in Lebanon and other countries in the region, such as Turkey, Egypt, and Saudi Arabia, tested the effects of combining colistin with select antimicrobial agents against colistin-resistant *A. baumannii*. The results of the studies were, in part, contradictory, which could be due to differences in the ST and AST profiles of the isolates used. In our study, we showed that the combination of colistin with either meropenem, ceftolozane–tazobactam, or teicoplanin had a synergistic effect on 100% of the tested isolates, while the colistin/levofloxacin combination showed synergistic activity for 87% of tested isolates. One of the two isolates on which the levofloxacin/colistin combination did not exhibit synergy (T31) exhibited different *pmrc* mutations from the rest of the samples, which could explain the variation. However, a possible explanation for such a variation in the other isolate (T36), which had the same *pmrc* mutation as the others, may reside in the presence and activation of specific efflux pumps. This is suggested by the high MIC value of colistin tested on this isolate. On the other hand, combining colistin with either tigecycline or amikacin did not have any effect. These results point to the importance of conducting tests to examine the effects of combining antimicrobials in each instance of a PDR *A. baumannii* infection due to the high disparity in the results, even amongst the studies performed in geographic proximity.

In our study, colistin MICs did not change after 7 days of serial passaging in LB broth, highlighting a degree of stability of the mutations responsible for colistin resistance in our *A. baumannii* isolates. It is worth noting that 87% of our isolates had similar growth rates compared to the wild-type strain *A. baumannii* DSM^®^ 30008, whereas 13% had lower growth rates. Hraiech et al. (2013) [27] reported that colistin resistance in their *A. baumannii* isolates was found to have been caused by mutations in *pmrA*, and that these isolates had higher growth rates compared to colistin-susceptible ones and the reference strain AYE [27]. Interestingly, Gerson et al. (2019) showed that the growth rate did not decrease in colistin-resistant clinical *A. baumannii* isolates that acquired resistance through mutations in the *pmrC* homolog *eptA* and a point mutation in *ISAba1* [28]. Our results also show that, in arguable congruence with the Gerson study, mutations in the *pmrC* variant are the reason for colistin resistance in 14 (93%) out of the 15 *A. baumannii* isolates, where 13/14 of them have unaffected growth rates compared to the reference strain (DSM^®^ 30008).

Furthermore, we reported in this study that the 15 colistin-resistant *A. baumannii* isolates belonged to Pasteur ST2 (*n* = 10) and ST187 (*n* = 5). The data related to ST187 are scarce in the literature; however, two studies from Lebanon investigated MDR and XDR *A. baumannii* isolates belonging mostly to ST2 and found them all to be susceptible to colistin [29,30]. In addition, a study on patients from the Middle East and North Africa (MENA) region performed in Germany found that all tested carbapenem-resistant *A. baumannii* isolates, including those belonging to ST2, were susceptible to colistin [31].

Our isolates harbored two variants of the *pmrC* gene, while multiple other genes and mutations conferring resistance to colistin were reported in the region, such as *mcr1*, *mcr2*, and *mcr3*, in addition to mutations in *pmrA*, *pmrB*, and *pmrC*. It is worth mentioning that the occurrence of the *pmrC* mutations in our study were independent of the MICs detected. This discrepancy could be explained by the possible presence of other unknown mechanisms conferring colistin resistance or complementing the function of the detected mutations. This observation underscores the necessity for antimicrobial susceptibility testing to confirm the activity of any resistance gene identified.

## 5. Conclusions

The gravity of the continuous increase in the occurrence of PDR pathogens prompts a global focus of efforts on addressing this challenge. One of the key elements is consistent surveillance using adequate tools, such as whole-genome sequencing, to improve our understanding of the molecular mechanisms of AMR, especially to colistin. It is crucial to consider antimicrobial resistance a prime concern in terms of the research, particularly on WHO priority pathogens, such as *A. baumannii*, to be able to generate innovative treatments in time to avoid the near-future projections of devastating outcomes on health and the economy worldwide.

## Figures and Tables

**Table 1:** MICs of tested antimicrobials for each isolate.

Isolates	MIC (µg/mL)															
	Colistin (R ≥ 4 µg/mL)	Zerbaxa	Amikacin (R ≥ 64 µg/mL)	Bactrim (R ≥ 4/76 µg/mL)	Levofloxacin (R ≥ 8 µg/mL)	Azithromycin	Teicoplanin	Tigecyclin (R ≥ 0.5 µg/mL)	Meropenem (R ≥ 8 µg/mL)	Imipenem	Gentamycin	Cefipeme	Tazocin	Ceftazidime	Ciprofloxacin	Tetracyclin
T11	4	64	>4096	64	16	256	256	64	128	256/128	>1024	256	512	128	64	1024
T19	512	64	128	32	16	64/32	1024	64	32	128	>1024	256	256	256	256/128	1024
T20	4	128	>4096	128	64	>256	8	64	32	64/128	>1024	512	512	512	128	1024
T31	512	8	128	16	8	128	32	16	8	16	8	64	128	128	64	512
T34	8	128	>4096	64	32	256	256	64	128	128	>1024	256	512	512/256	64	1024
T36	512	128	>4096	8	32	256	128	16	16	16	>1024	128	256	512	128	1024
T38	4	128	>4096	128	32	256	128	64	128	128	1024	256	1024	512	64	1024
T39	4	128	>4096	64	32	>256	256	32	256	128	>1024	512	1024	512	64	1024
T40	8	128	>4096	64	32	>256	256	32	128	64	>1024	265	512	512	128	1024
T47	4	512	>4096	64	32	>128	512	64	128	128	>2048	512	256	512	64	1024
T48	4	64	>4096	64	32	>128	512	64	128	64	>2048	128	512	256	64	1024
ACN1	512	128	>4096	32	16	256	64	32	64	256	>10,204	256	512	512	64	1024
ACN2	64	16	>4096	64	64	>256	128	32	128	128	>1024	128	512	256	128	1024
ACN3	512	16	>4096	32	32	8	128	64	8	16	2	128	256	256	128	1024
ACN4	1	128	>4096	1	64	>256	256	64	128	64	>1024	128	256	512	128	
ACN5	512	16	>4096	64	16	>256	32	64	64	128	1024	256	512	512	64	1024

**Table 2:** Antimicrobial-resistance genes detected in each isolate.

Isolate	Aminoglycosides	Sulphon-Amides	Tetra-Cyclines	Macrolides	Beta-Lactams
T11	aph(3″)-Ib; aph(6)-Id; armA; aph(3′)-Ia	sul2	tet(B)	mph(E); msr(E)	OXA-66; OXA-23; ADC-25; TEM-1D
T19	aph(3″)-Ib; aph(6)-Id; armA; aph(3′)-Ia	N/A	tet(B)	mph(E); msr(E)	OXA-66; OXA-23; ADC-25; TEM-1D
T20	aph(3″)-Ib; aph(6)-Id; armA; aph(3′)-Ia	N/A	tet(B)	mph(E); msr(E)	OXA-66; OXA-23; ADC-25; TEM-1D
T31	armA; aph(3′)-Ia	N/A	N/A	mph(E); msr(E)	OXA-66; blOXA-23; ADC-25; TEM-1D
T34	aph(3″)-Ib; aph(6)-Id; armA; aph(3′)-Ia	N/A	tet(B)	mph(E); msr(E)	OXA-66; OXA-23; ADC-25; TEM-1D
T36	aph(3″)-Ib; aph(6)-Id; armA; aph(3′)-Ia	N/A	tet(B)	mph(E); msr(E)	OXA-66; OXA-23 ADC-25; TEM-1D
T38	aph(3″)-Ib; aph(6)-Id; armA; aph(3′)-Ia	N/A	tet(B)	mph(E); msr(E)	OXA-66; OXA-23; ADC-25; TEM-1D
T39	aph(3″)-Ib; aph(6)-Id; aph(3′)-VIa	sul2	tet(B)	N/A	OXA-66; OXA-23; ADC-25
T40	aph(3″)-Ib; aph(6)-Id; armA; aph(3′)-Ia	sul2	tet(B)	mph(E); msr(E)	OXA-66; OXA-23; ADC-25; TEM-1D
T47	armA; aph(3′)-Ia; aph(3″)-Ib; aph(6)-Id; aph(3′)-VIa	sul2	tet(B)	mph(E); msr(E)	OXA-66; OXA-225; ADC-25; TEM-1D
T48	aph(3″)-Ib; aph(6)-Id; armA; aph(3′)-Ia	sul2	tet(B)	mph(E); msr(E)	OXA-66; OXA-23; ADC-25; TEM-1D
ACN1	armA; aph(3′)-Ia; aph(3″)-Ib; aph(6)-Id; aph(3′)-VIa	sul2	tet(B)	mph(E); msr(E)	OXA-66; OXA-23; ADC-25; TEM-1D
ACN2	armA; aph(3′)-Ia; aph(3″)-Ib; aph(6)-Id; aph(3′)-VIa	sul2	tet(B)	mph(E); msr(E)	OXA-66; OXA-23; ADC-25; TEM-1D
ACN3	armA; aph(3′)-Ia; aph(3″)-Ib; aph(6)-Id; aph(3′)-VIa	sul2	tet(B)	mph(E); msr(E)	OXA-66; OXA-23; ADC-25; TEM-1D
ACN5	armA; aph(3′)-Ia; aph(3″)-Ib; aph(6)-Id; aph(3′)-VIa	sul2	tet(B)	mph(E); msr(E)	OXA-66; OXA-23; ADC-25; TEM-1D

**Table 3:** Molecular investigation results of colistin resistance in the 15 *A. baumannii* isolates.

Isolate Code	Colistin MIC (µg/mL)	Resistance Determinant	Mutations
T11	4	pmrC variant	G326A and R109H
T19	512	-	-
T20	4	pmrC variant	G326A and R109H
T31	512	pmrC variant	C950T and A317V
T34	8	pmrC variant	G326A and R109H
T36	512	pmrC variant	G326A and R109H
T38	4	pmrC variant	G326A and R109H
T39	4	pmrC variant	G326A and R109H
T40	8	pmrC variant	G326A and R109H
T47	4	pmrC variant	G326A and R109H
T48	4	pmrC variant	G326A and R109H
ACN1	512	pmrC variant	G326A and R109H
ACN2	64	pmrC variant	G326A and R109H
ACN3	512	pmrC variant	G326A and R109H
ACN5	512	pmrC variant	G326A and R109H

-: Not detected.

## Data Availability

All sequences were deposited in the NCBI under project number PRJNA979211.

## Supplementary Materials

The following supporting information can be downloaded at: https://www.mdpi.com/article/10.3390/microorganisms12020349/s1, Figure S1: Growth rate assay results of isolates T11, T20, T34, and T36 compared to *A. baumannii* DSM 30008; Figure S2: Growth rate assay results of isolates T38, T39, T40, and T47 compared to *A. baumannii* DSM 30008; Figure S3: Growth rate assay results of isolates T48, ACN1, ACN3, and ACN5 compared to *A. baumannii* DSM 30008; Figure S4: Growth rate assay results of isolate T31 compared to *A. baumannii* DSM 30008.
