# Supplementary figures and images for: Molecular Characteristics of Colistin Resistance in Acinetobacter baumannii and the Activity of Antimicrobial Combination Therapy in a Tertiary Care Medical Center in Lebanon
Source: Microorganisms. 2024 Feb 8;12(2):349. doi: 10.3390/microorganisms12020349 (PMC10892383; doi:10.3390/microorganisms12020349)

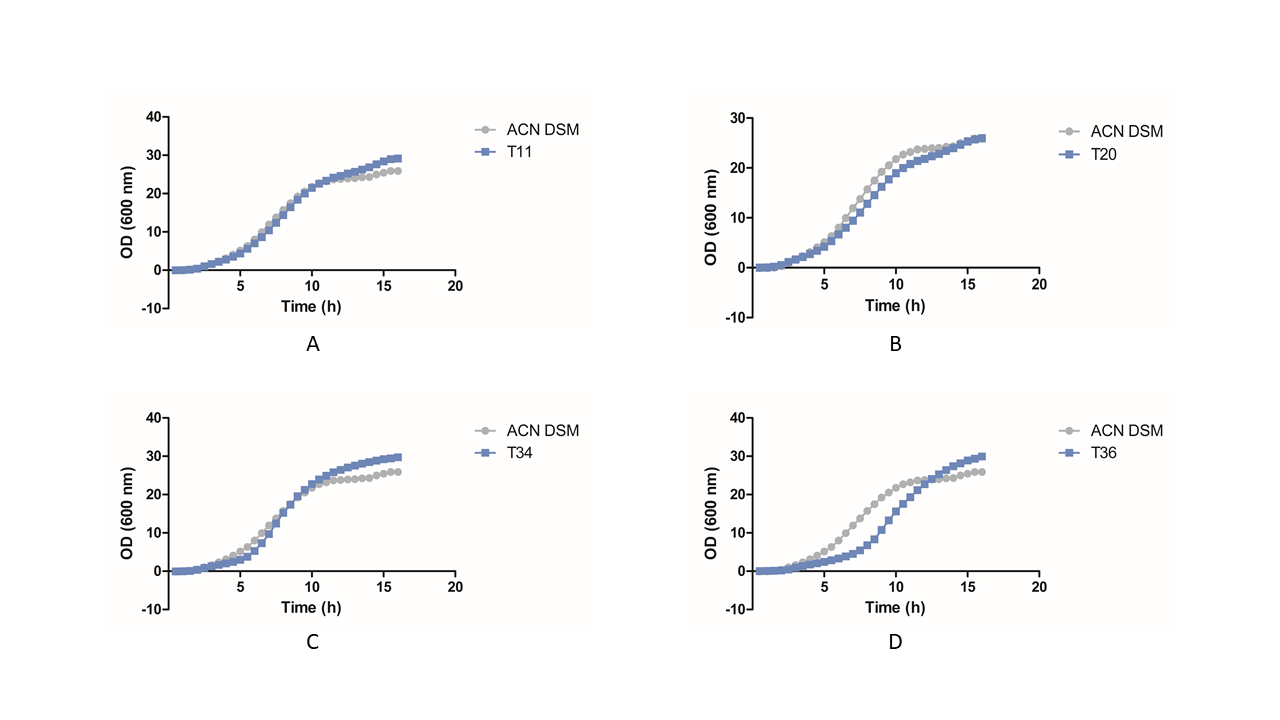

Supplement: Supplementary file 1 [file microorganisms-12-00349-s001.zip › Figure S1.tif]

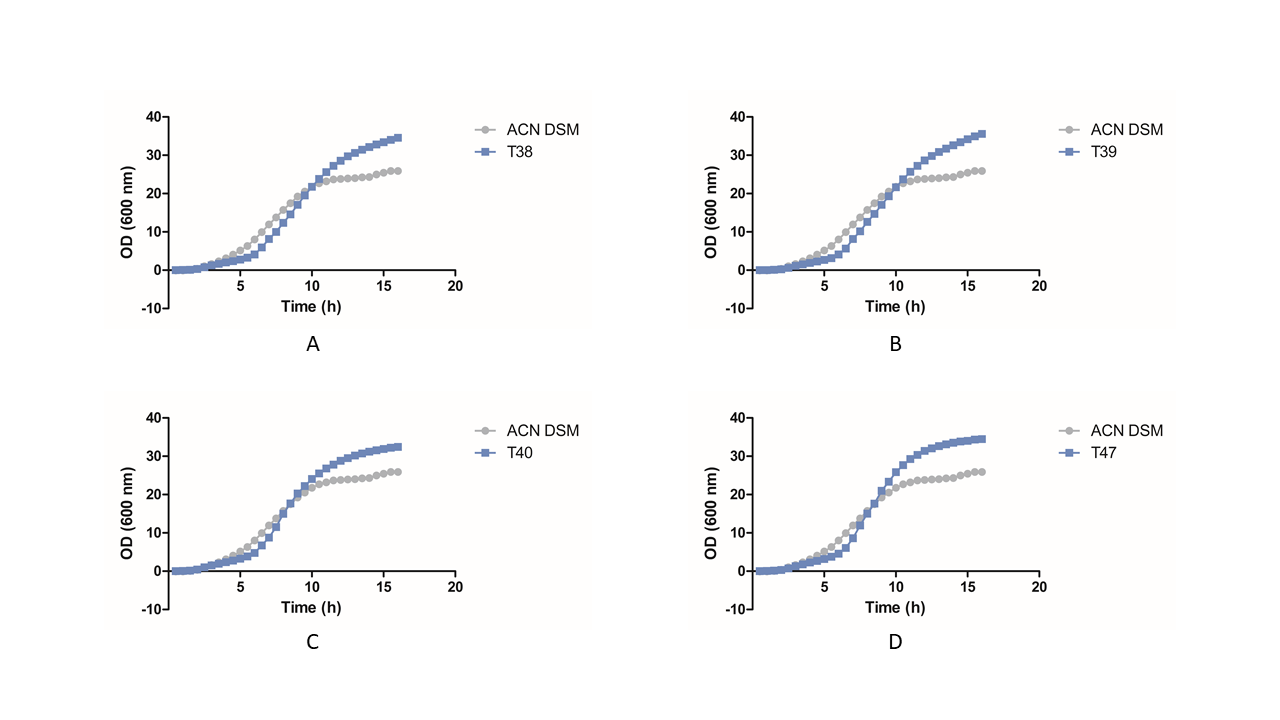

Supplement: Supplementary file 1 [file microorganisms-12-00349-s001.zip › Figure S2.tif]

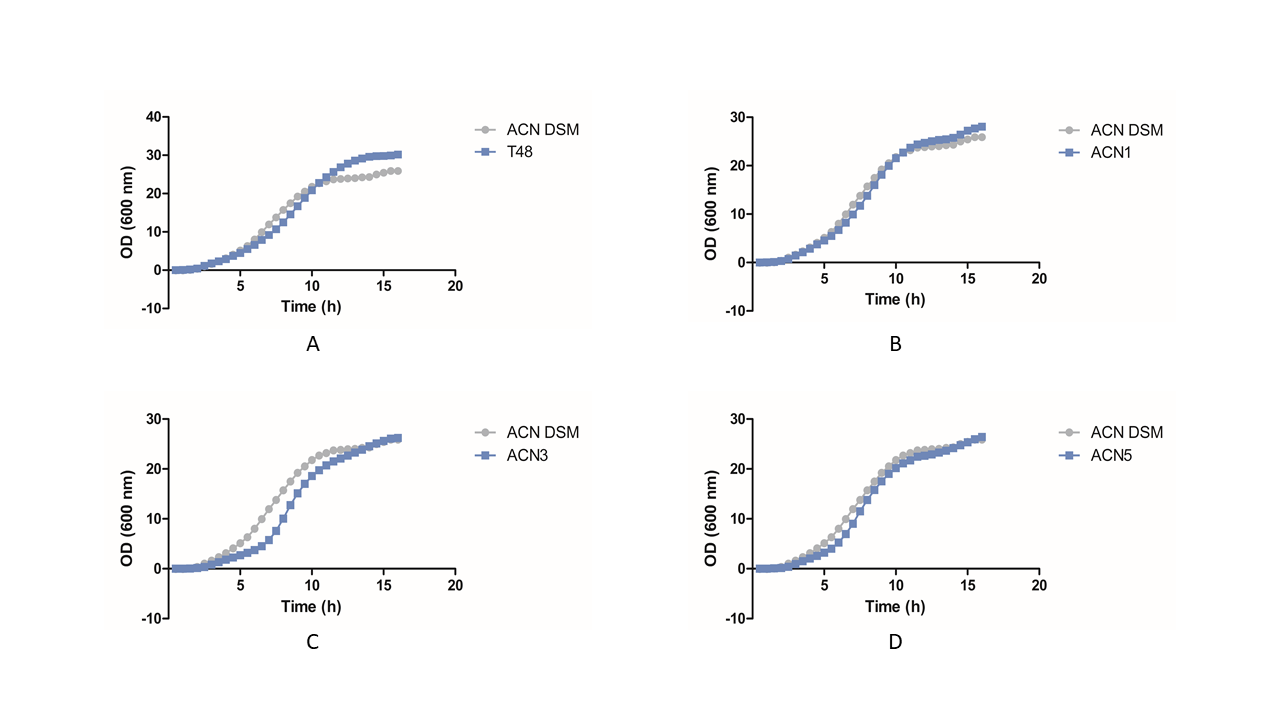

Supplement: Supplementary file 1 [file microorganisms-12-00349-s001.zip › Figure S3.tif]
